# Perspectives of students and mentors on a formal mentorship program in Saudi Arabia

**DOI:** 10.5116/ijme.587a.06f8

**Published:** 2017-01-23

**Authors:** Maria Ghawji, Muhammad Raihan Sajid, Abdul Ahad Shaikh, Rimsha Sheriff, Peter Cahusac, Khaled Alkattan

**Affiliations:** 1 College of Medicine, Alfaisal University, Riyadh, Saudi Arabia

**Keywords:** Students and mentors, formal mentorship program, Saudi Arabia

## Introduction

Student mentorship is a dynamic reciprocal relationship between an advanced career incumbent (mentor) and a beginner (protégé), aimed at promoting the development of both.^1^ The main goals of any mentorship program include career counselling and development alongside professional enhancement.^2^^-^^4^

While numerous studies highlight the importance of a formal mentorship program at the undergraduate level, no significant reports have emerged from the Kingdom of Saudi Arabia regarding such structured programs. There remains a need to identify and evaluate gaps in the efficacy of mentorship programs to establish and measure the effectiveness of such programs in undergraduate medical education. The aim of this perspective is to describe the strengths, weaknesses and challenges of the mentorship program among preclinical medical students at Alfaisal University, Riyadh.

### Program organization

At Alfaisal University a formal mentorship program was introduced in 2012 whereby students were assigned a mentor at the beginning of his/her first year. The same mentor was maintained through the student’s tenure at the University. The program aimed to cover academics, career plans and personal growth. Students were provided with the name, email, office location, contact extension, and office hours. The student mentors were also notified about the program. In a separate email, mentors were advised regarding their responsibilities, and are asked to contact their mentees to arrange an initial meeting. Mentees had the option to change their mentors if they were not satisfied and had to provide a reasonable rationale for this change.

### Challenges and solutions

In the beginning, there were challenges to the program from both mentee and mentors. There was initial resistance to the idea and a lack of trained mentors. To overcome this challenge an introduction to the program was arranged for both mentors and mentees separately. In the faculty meeting, the program organization was discussed with all faculty and expectations from the mentors was highlighted. The college dean and program director stressed the importance of mandatory meetings with mentees, and the importance of documenting these meetings. Mentors were told to take a proactive role in this relationship. Any queries and questions were addressed in the meeting. The faculty was required to display office hours (dedicated time slots) for mentees on their office doors and also to communicate this information to their mentees.

An introductory email to the students also explained the scope of the program and responsibilities of mentees.

### Identification of weak students and academic counselling

The faculty was provided access via ‘faculty web access’ to the student grades so that they could track student progress throughout the semester and identify weak students who required counselling. As part of the mentorship program, such weak students were offered academic counselling and teaching tips for improvement in their performance. 

### Peer teaching

One of the unique features of this program has been the introduction of peer teaching. Senior students were recruited to coach and teach the weak students on a one-to-one basis. Senior students were compensated for their time and effort with a stipend.

### Student development committee

As part of the efforts to improve the mentorship program, a student development committee was also formed with representatives from senior faculty and students. The scope of the committee included overseeing summer research internships, clinical electives both locally and abroad, and students’ research-related activities during the academic year, e.g. conferences, symposia, workshops and research papers.

### Mentee perspective

To assess the success of the program, we delivered a survey to mentees requiring them to inform us about their perception of the program. Some of the findings of the survey showed that many of the mentees never had a single meeting with their mentor. Almost 60% of the mentees opined that mentors were supportive. Overall, the best satisfaction level was seen in the responses of third-year medical students.

Exploratory statistical analyses found no differences between male and female responses to the Likert-scaled items in the questionnaire. Similar analyses looking across the three years indicated that year three students were more positive compared with other years, notably on some items. These items are: “my mentor is approachable”, “my mentor is supportive and encouraging”, “my mentor motivates me to improve my work”, “my mentor provides constructive critiques”, “my mentor is helpful in providing direction and guidance”.

Almost half the surveyed students believed that the mentorship program was helpful in their professional growth and grade improvement. When asked about possible mechanisms to improve the overall effectiveness of the program, the suggestions included better communication initiated by mentors, such as regular meetings. Most of the students identified lack of motivation as the major hurdle in the program.

### Mentor Perspective

Feedback was also collected from the faculty members. Most mentors reported that mentee response to an email was within hours. Approximately 75% of the mentors were willing to spend 1-2 hours per week on the program. The majority of mentors claimed to have met their mentees, and checked on their mentee's progress once every other month. Approximately half thought that students did not accept criticism, and failed consequently to reassess their performance. The final section of the survey included an open-ended question similar to that given to the mentees about possible improvements. The two most common suggestions were that the mentors should be provided with assessment information for mentee exams and that mandatory meetings should be scheduled alongside appropriate faculty training. 

## Discussion

Studies have emphasized the importance of developing mentoring skills among both experienced and inexperienced mentors. Indeed, studies indicate that mentors of most programs are not adequately trained or provided with sufficient guidelines.^5^^-^^8^ We have worked through meetings and electronic communications to ensure that both mentors and mentees are informed of their responsibilities and the scope of their relationship. Therefore it is of paramount importance to provide mentors with the chance of acquiring mentoring skills.

We suggest that specific mentor training is required to facilitate productive mentor/mentee relationships. Similarly, students should be informed and familiarized about their role as mentees. We have seen significantly greater harmony between mentors and third-year students, where one could argue that third-year students had been subject to passive training for the longest, and at that stage had received sufficient training to be ‘good mentees’. 

We suggest that while mentor training is important, it is more important that students develop a positive attitude towards the mentorship program early in their tenure, rather than rely on this developing over three years. It is clear that the majority of students in their first and second years do not appreciate the importance of their relationship with their mentors, or what they should be extracting from this relationship. Perhaps this explains the increased responsiveness of the third year students to mentor feedback compared to the other two years.

## Conclusions

Being a newly established program, we have faced many challenges to make it acceptable to both mentors and mentees. We believe that the introduction of newer measures like peer-to-peer teaching and mentoring, structured meetings, more research opportunities to the mentees and developing a cordial but professional long term relationship, will help us in overcoming the challenges. We suggest that communication and motivation, on the part of both mentor and mentee, are the most important characteristics for the success of the program. Further success may depend on training workshops and instruction sessions about the mentorship program given to both faculty mentors and first-year student mentees. While issues remain to be resolved, the program has great potential to deliver benefits to students in the longer term.

### Conflict of Interest

The authors declare that they have no conflict of interest.
